# Intraneural Melanoma Identified With MART-1 Immunostaining in Mohs Micrographic Surgery

**DOI:** 10.7759/cureus.58920

**Published:** 2024-04-24

**Authors:** S. Caleb Freeman, Nicole N Dacy, Brett C Neill, Cary Chisholm, Stanislav N Tolkachjov

**Affiliations:** 1 Dermatology, Oregon Health & Science University, Portland, USA; 2 Dermatology, Baylor Scott & White Medical Center, Temple, USA; 3 Dermatology and Mohs Micrographic Surgery, Swann Dermatology, Springfield, USA; 4 Dermatopathology, Epiphany Dermatology, Waco, USA; 5 Mohs Micrographic Surgery, Epiphany Dermatology, Dallas, USA

**Keywords:** immunohistochemistry (ihc), mart-1 immunostaining, mohs for melanoma, mohs micrographic surgery, neurotropic melanoma, intraneural melanoma

## Abstract

Mohs micrographic surgery (MMS) utilizing melanoma antigen recognized by T-cells (MART-1) immunostaining is an increasingly common method of treatment for minimally invasive melanoma in anatomically constrained areas such as the face, ears, or acral sites. Neurotropic melanoma, also known as neurotrophism in melanoma, refers to the invasion of melanoma cells into the nerves. As such, these tumors can extend well beyond anticipated clinical tumor margins which can increase the risk of local recurrence. Here, we present a case of neurotropic melanoma successfully identified during MMS using MART-1 immunostaining, which was then confirmed with permanent sectioning.

## Introduction

Despite controversy over the surgical treatment of cutaneous melanomas, Mohs micrographic surgery (MMS) has been shown to be an effective treatment for in situ and minimally invasive melanoma with improved survival outcomes [1,2]. Low recurrence rates have been demonstrated in MMS utilizing melanoma antigen recognized by T-cells (MART-1) immunostaining [2,3]. Compared to traditional excision, MMS offers the advantage of examining 100% of the surgical margins to confirm tumor clearance while sparing as much healthy tissue as possible. We present a case of minimally invasive melanoma being treated with MMS in which intraneural melanoma was identified with MART-1 immunostaining. Intraneural melanoma, also known as melanoma with neurotrophism, is a rare occurrence, reported to occur in less than 2% of all melanoma cases [4].

## Case presentation

A 77-year-old Caucasian male was referred for MMS for definitive treatment of minimally invasive melanoma with a Breslow thickness of at least 0.2 mm. Histology of the first stage showed brown pigment within several nerves on MART-1 and hematoxylin and eosin (H&E) staining. A second stage was taken to clear the peripheral melanoma in situ. While peripheral margins had been cleared, concern persisted for intraneural invasion given the unexplained intraneural pigmentation. A pathologist was consulted remotely to assess frozen histologic sections for neurotropism with intraneural invasion, a finding that had not been reported in the referral documentation, and recommended permanent sectioning for further analysis. After reviewing these findings with the patient, a discussion was held regarding the option for delaying the repair of the surgical site while waiting for permanent section analysis. Sentinel lymph node biopsy was also discussed. The patient ultimately elected for immediate repair without sentinel node biopsy. The patient’s defect was then repaired with a dorsal nasal rotation flap; however, care was taken to avoid any additional alteration to the deep margin as well as any disturbance of surrounding margin tissue in case permanent sections showed neurotropism in melanoma. Given the rarity of finding intraneural melanoma during MMS, the slides, the debulk specimen, and the Mohs sections were sent for permanent sectioning.

**Figure 1:**
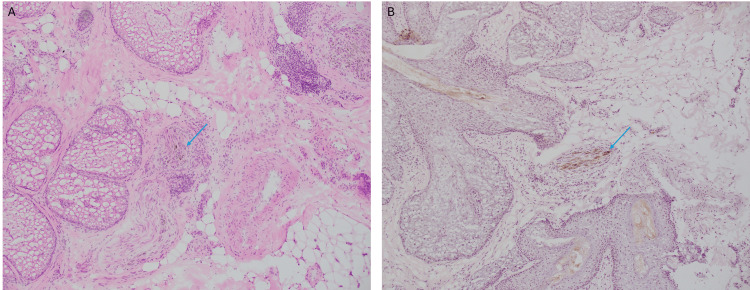
Permanent sections reviewed by dermatopathology showed intraneural melanoma. A. Hematoxylin and eosin stained section demonstrating pigment, denoted by blue arrows, within the nerve (10x). B. Immunohistochemical staining with melanoma antigen recognized by T cells 1 (MART-1) confirmed the presence of melanocytes, denoted by blue arrows, within the nerve (10x).

The permanent sections were reviewed by dermatopathology which confirmed neurotropism of melanoma. These findings were identified with H&E and MART-1 immunostaining of fixed histologic sections (Figures 1A, 1B).

**Figure 2:**
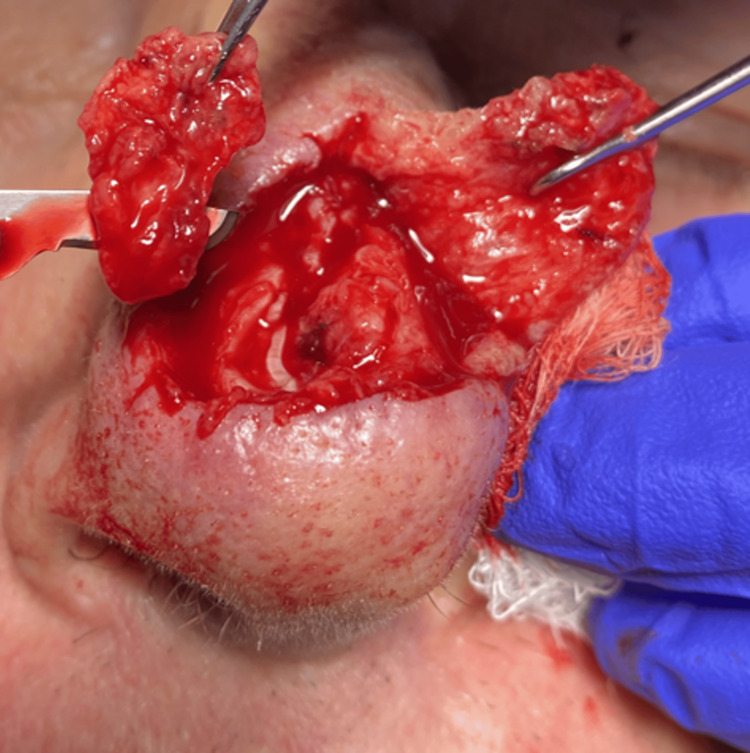
Re-excision of the Mohs micrographic surgery defect. Elevation of the dorsal nasal rotation flap tip and subsequent excision of the entire base.

The patient was asked to return to the clinic. The distal portion of the flap was re-elevated. After a comparison of the defect photograph and the surgical site after flap re-elevation, additional stages were taken around the entire original defect with a depth to ensure adequate tissue removal to avoid a false negative (Figure 2).

**Figure 3:**
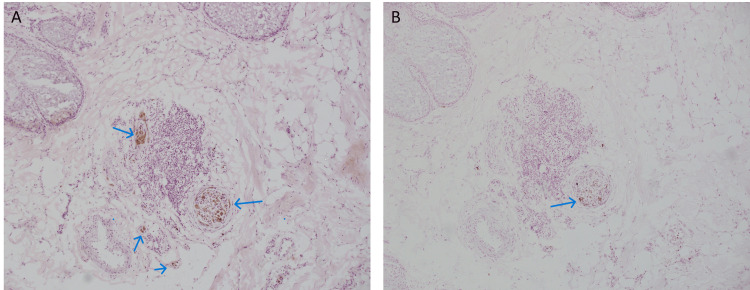
Intraneural and perineural pigment identified on frozen sections during Mohs micrographic surgery. A. Intraneural and perineural pigment, denoted by blue arrows, identified during Mohs micrographic surgery with hematoxylin and eosin stain (10x magnification). B. Comparative section with melanoma antigen recognized by T cells 1 (MART-1) immunostaining with intraneural and perineural melanocytes as denoted by the blue arrow (10x magnification).

Intraneural melanoma was again identified at this stage (Figures 3A, 3B). Ultimately, three stages were required to clear the melanoma. He was subsequently referred to the medical and radiation oncology tumor board for further discussion of adjunctive treatment options.

**Figure 4:**
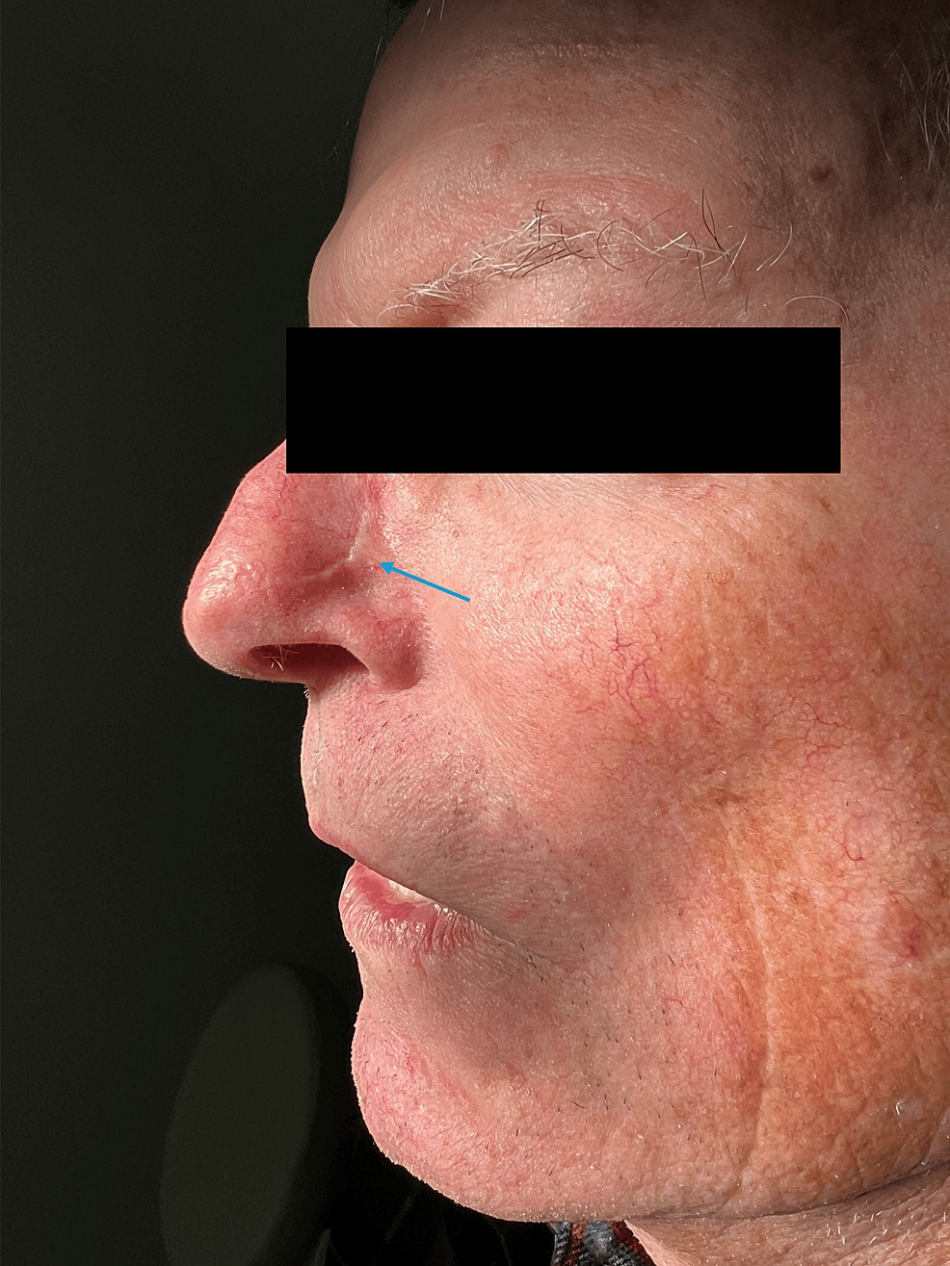
Surgical site at three-year follow up. An exam showed a well-healed scar (blue arrow) without pigment or nodularity.

The patient was seen for a three-year follow-up with an acceptable cosmetic outcome (Figure 4) and no evidence of local, regional, or distant recurrence of his melanoma. 

## Discussion

Neurotropism is defined as perineural or intraneural invasion of tumor cells and is usually associated with desmoplastic melanoma [5]. However, while less frequent, it has been shown to be associated with other melanoma subtypes [6]. Neurotropism in melanoma can extend well beyond the edge of the primary tumor bulk, as was the case in our patient. This can increase the risk of local recurrence if surgical margins are not completely cleared [6].

Studies have shown lower recurrence rates and improved survival outcomes for melanomas treated with MMS when compared to wide local excision [1]. This case demonstrates the usefulness of MART-1 in identifying neurotropism in patients undergoing MMS. We also highlight the need to closely evaluate perineural and intraneural involvement in addition to deep margins in cases of melanoma while performing MMS. While Mohs surgeons rarely consult dermatopathologists during MMS, permanent section analysis may be helpful in select cases, such as concern for intraneural melanoma. In this case, it would have been beneficial to delay reconstruction until permanent sections resulted; however, the patient had a strong preference to repair the defect immediately, even after reviewing the risks and benefits of immediate repair. Lastly, this case epitomizes how MMS with immunohistochemistry allows surgeons to identify and track melanoma, even with neurotropism, in real-time. 

## Conclusions

Mohs surgeons should be aware of the potential for identifying neurotropism in melanoma during Mohs micrographic surgery (MMS). Immunohistochemical staining with MART-1 can help identify intraneural invasion. Permanent section analysis following MMS can be a useful tool in cases with intraneural melanoma. Delayed repair should be considered in any cases which require permanent section analysis. This case demonstrates the importance of Mohs surgeons closely collaborating with dermatopathologists when challenging findings are identified during MMS.
